# *HFE* mRNA expression is responsive to intracellular and extracellular iron loading: short communication

**DOI:** 10.1007/s11033-017-4123-2

**Published:** 2017-08-24

**Authors:** Kosha J. Mehta, Sebastien Farnaud, Vinood B. Patel

**Affiliations:** 1 Department of Biomedical Sciences, University of Westminster, 115 New Cavendish Street, London, W1W 6UW UK; 2 School of Life Sciences, Coventry University, 138 James Starley Building, Coventry, CV1 5FB UK

**Keywords:** HFE, Iron-sensing, Holotransferrin, Hepcidin

## Abstract

In liver hepatocytes, the *HFE* gene regulates cellular and systemic iron homeostasis by modulating cellular iron-uptake and producing the iron-hormone hepcidin in response to systemic iron elevation. However, the mechanism of iron-sensing in hepatocytes remain enigmatic. Therefore, to study the effect of iron on *HFE* and hepcidin *(HAMP*) expressions under distinct extracellular and intracellular iron-loading, we examined the effect of holotransferrin treatment (1, 2, 5 and 8 g/L for 6 h) on intracellular iron levels, and mRNA expressions of *HFE* and *HAMP* in wild-type HepG2 and previously characterized iron-loaded recombinant-TfR1 HepG2 cells. Gene expression was analyzed by real-time PCR and intracellular iron was measured by ferrozine assay. Data showed that in the wild-type cells, where intracellular iron content remained unchanged, *HFE* expression remained unaltered at low holotransferrin treatments but was upregulated upon 5 g/L (p < 0.04) and 8 g/L (p = 0.05) treatments. *HAMP* expression showed alternating elevations and increased upon 1 g/L (p < 0.05) and 5 g/L (p < 0.05). However, in the recombinant cells that showed higher intracellular iron levels than wild-type cells, *HFE* and *HAMP* expressions were elevated only at low 1 g/L treatment (p < 0.03) and were repressed at 2 g/L treatment (p < 0.03). Under holotransferrin-untreated conditions, the iron-loaded recombinant cells showed higher expressions of *HFE* (p < 0.03) and *HAMP* (p = 0.05) than wild-type cells. *HFE* mRNA was independently elevated by extracellular and intracellular iron-excess. Thus, it may be involved in sensing both, extracellular and intracellular iron. Repression of *HAMP e*xpression under simultaneous intracellular and extracellular iron-loading resembles non-hereditary iron-excess pathologies.

**Electronic supplementary material:**

The online version of this article (doi:10.1007/s11033-017-4123-2) contains supplementary material, which is available to authorized users.

## Introduction

Maintenance of cellular and systemic iron homeostasis in the body is a dynamic process involving several signal transduction pathways. The haemochromatosis protein HFE maintains body iron homeostasis by participating in the induction of hepcidin (*HAMP*), the systemic iron regulator, by a yet incompletely understood mechanism [1–3]. Mutations in the genes *HFE* or *HAMP* causes diminished hepcidin production which results in systemic and tissue iron overload, referred as hereditary haemochromatosis [4]. However, despite the presence of functional wild-type alleles of these genes, low to moderate tissue iron excess is also observed in non-hereditary conditions such as alcoholic liver disease, hepatitis C infections, non-alcoholic fatty liver disease, non-alcoholic steato hepatitis and type 2 diabetes [5–8]. In these cases, iron loading can exacerbate the pathophysiology via excess-iron-induced oxidative stress [9]. Thus, it is important to fully delineate their iron-sensing mechanisms to formulate therapeutic interventions, particularly for the low-moderate iron-loaded conditions where, unlike hereditary hemochromatosis, phlebotomy is not practiced for removal of excess iron.

The mRNA response of *HFE* to increasing extracellular and intracellular iron and its relationship with *HAMP* expression at transcript level have not been studied so far. Hence, in this short study, we investigated the effect of a range of holotransferrin (holo-Tf) concentrations (1–8 g/L) on *HFE* and *HAMP* mRNA expressions, and intracellular iron content. First, we observed these responses in the wild type (Wt) HepG2 cells, where holo-Tf supplementation represent physiological conditions with extracellular (systemic) iron elevation prior to intracellular/tissue iron loading. Then, we examined the responses in the previously characterized recombinant (rec)-TfR1 HepG2 cells [10]. As these cells can achieve intracellular iron overloading [10], holo-Tf supplementation to these cells represent pathological conditions, which show simultaneously increased extracellular (systemic) and intracellular iron levels. Finally, to understand the exclusive effect of high intracellular iron content, we compared the expression levels between holo-Tf-untreated Wt and recombinant cells. Unlike most previous holo-Tf supplementation studies that were conducted at longer time-points of 24, 48 or 72 h [11–13], here, we studied the effect following 6 h of holotransferrin treatment to examine early responses.

## Materials and methods

### Cell culture and treatments

Maintenance of cells and holo-Tf supplementation to the Wt HepG2 cells (Health Protection Agency, UK) and rec-TfR1 HepG2 cells was as described previously [10]. Cells were treated with holo-Tf (1, 2, 5 and 8 g/L) prepared in serum-free EMEM (0 g/L) for 6 h and assessed for various parameters. As 8 g/L holo-Tf represent a very high concentration and the rec-TfR1 HepG2 cells had the potential for intracellular iron-overloading following holo-Tf supplementation [10], the effect of this concentration was studied only in Wt cells.

### Determination of intracellular iron content

Cellular iron content determined by ferrozine assay [14] was normalized to protein, content, as quantified by Bradford method. Iron levels were expressed as nmoles iron/mg protein.

### Gene expression analysis

Primers (Invitrogen, UK) for expression analyses, RNA extraction, cDNA conversion and assessment for mRNA expression via real-time PCR by using Quantifast SYBR green kit (Qiagen, UK), was as previously described [10, 15]. Data was analyzed by the relative quantification method, Delta–Delta C_t_ (∆∆Ct) and expressed as 2^−∆∆Ct^ [16].

### Statistical analysis

Data analysis was performed using one-way ANOVA. The level of significance was set at p < 0.05. Data was presented as mean ± SEM (n = 3).

## Results

**Fig. 1:**
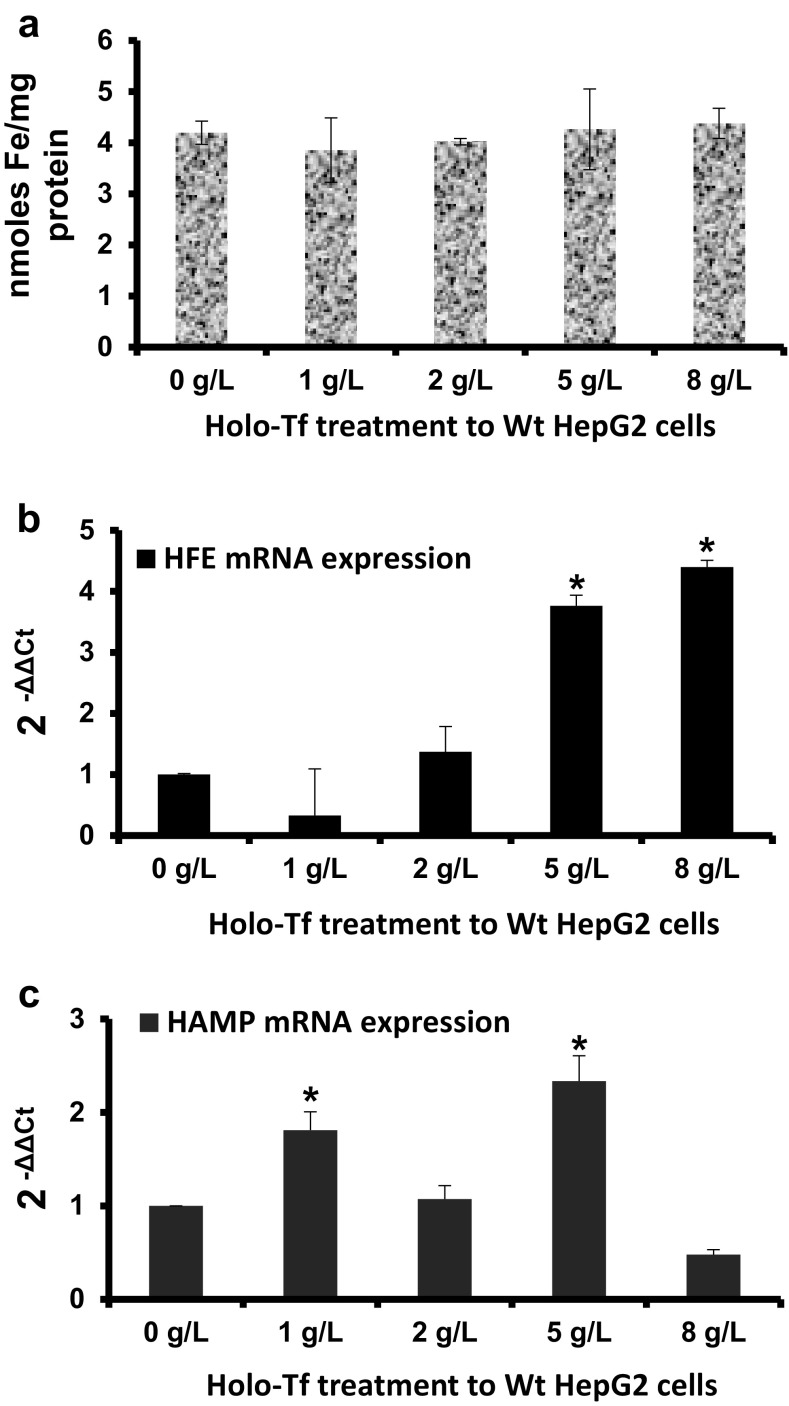
Effects of holo-Tf supplementation in Wt HepG2 cells. Wt HepG2 cells were treated with holo-Tf for 6 h. Following the treatment, intracellular iron levels were measured and expressed per mg protein (**a**). *HFE* (**b**) and *HAMP* (**c**) mRNA expressions was assessed and expressed relative to untreated (0 g/L) cells. Data is presented as mean ± SEM (n = 3). *p ≤ 0.05 compared to untreated (0 g/L) controls

In the Wt cells, while intracellular iron content remained unaltered following holo-Tf supplementation (Fig. 1a), *HFE* mRNA expression significantly increased by 3.5-fold (p < 0.04) upon 5 g/L treatment and further increased by 4.5-fold (p = 0.05) upon 8 g/L treatment (Fig. 1b). Expression levels remained unaltered at lower concentrations of 1 and 2 g/L (Fig. 1b). Differentially, *HAMP* expression showed a pattern of alternating responses i.e. a significant 1.8-fold (p < 0.05) up-regulation upon 1 g/L treatment, unaltered expression upon 2 g/L treatment followed by a significant 2.3-fold up-regulation upon 5 g/L treatment (p < 0.05) and then, down-regulation upon 8 g/L treatment (Fig. 1c).

**Fig. 2:**
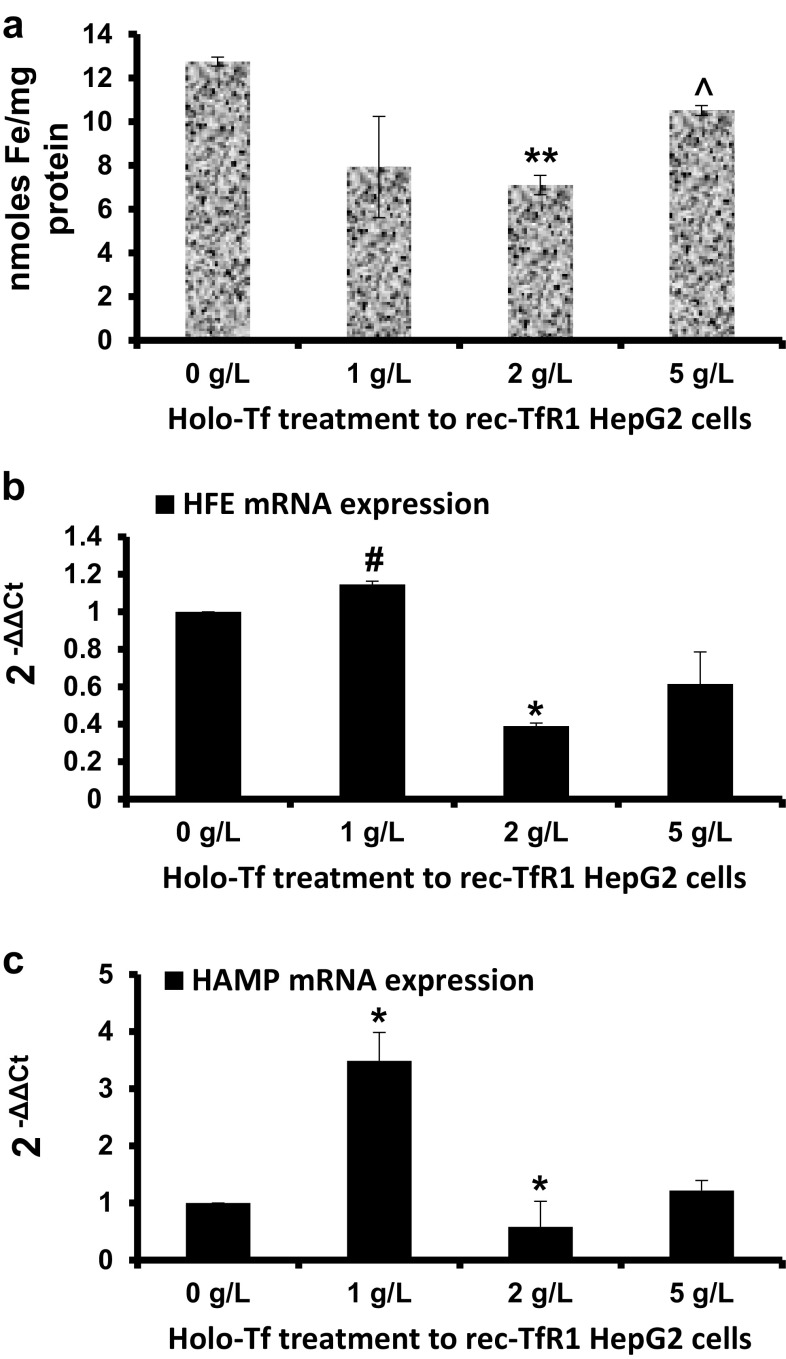
Effects of holo-Tf supplementation in rec-TfR1 HepG2 cells. Rec-TfR1 HepG2 cells were treated with holo-Tf for 6 h. Following the treatment, intracellular iron levels were measured and expressed per mg protein (**a**). *HFE* (**b**) and *HAMP* (**c**) mRNA expressions was assessed and expressed relative to untreated (0 g/L) cells. Data is presented as mean ± SEM (n = 3). *p < 0.03, **p < 0.01 and ^#^p = 0.07 compared to untreated (0 g/L) controls. ^p < 0.03 compared to 2 g/L treatment

Prior to expression studies in the recombinant cells, intracellular iron loading was confirmed. Data showed that following treatment with most holo-Tf concentrations, intracellular iron content in these cells was higher than Wt cells (Figs. 1a, 2a). In the recombinant cells, over the increasing holo-Tf concentrations, although intracellular iron content decreased at 2 g/L (p < 0.01), it increased at 5 g/L treatment (p < 0.03) that restored the high levels, as in untreated conditions (Fig. 2a). These cells differed from the Wt cells in *HFE* and *HAMP* expression patterns. Here, *HFE* expression increased upon 1 g/L (p = 0.07), but then decreased upon 2 g/L holo-Tf treatment (p < 0.03), and remained unaltered at 5 g/L (Fig. 2b). Similarly, *HAMP* expression increased by 3.5-fold at 1 g/L (p < 0.03) followed by a repression at 2 g/L (p < 0.03)and remained unaltered at 5 g/L treatment (Fig. 2c).

**Fig. 3:**
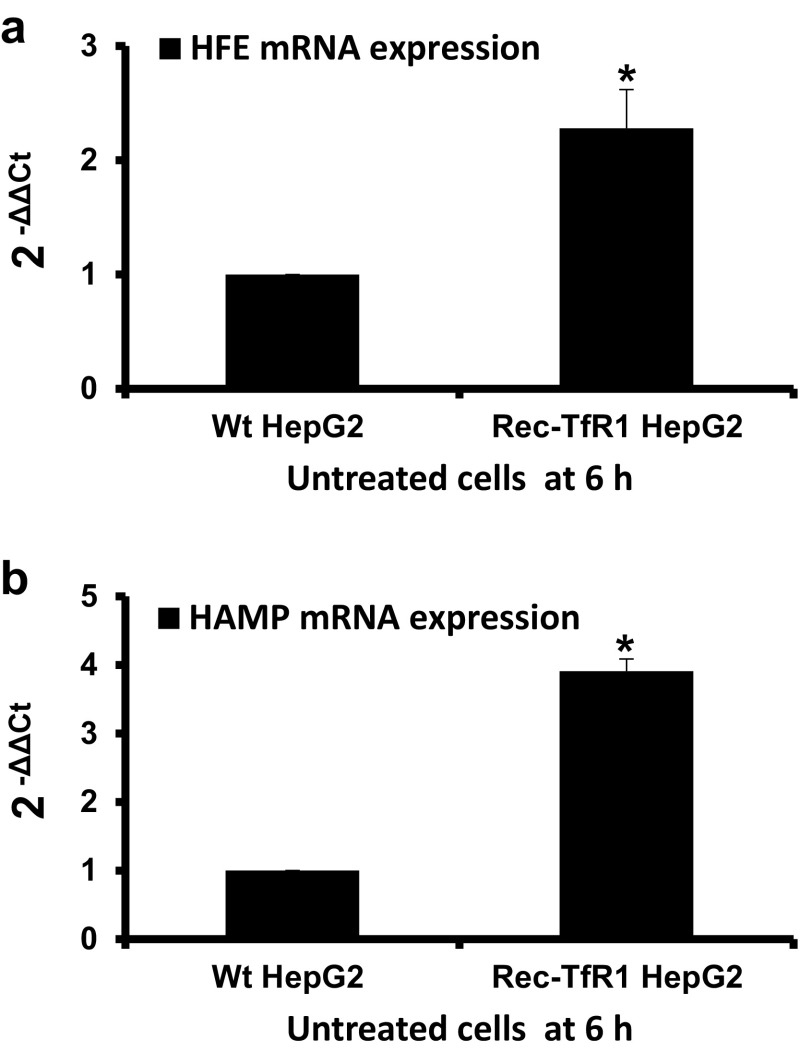
*HFE* and *HAMP* mRNA expressions in rec-TfR1 HepG2 cells relative to Wt HepG2 cells. The mRNA expressions of *HFE* (**a**) and *HAMP* (**b**) in the recombinant cells were expressed relative to Wt HepG2 cells under untreated conditions at the 6 h time point. Data is presented as mean ± SEM (n = 3). *p ≤ 0.05 compared to Wt HepG2 cells

Further, to understand the exclusive effect of intracellular iron loading, *HFE* and *HAMP* expressions in untreated cells were compared. Data showed that the recombinant cells expressed higher levels of *HFE* and *HAMP* mRNA than Wt cells (2.3-fold; p < 0.03 and 3.9-fold; p = 0.05, respectively) (Fig. 3a, b).

## Discussion

The genes *HFE* and *HAMP* are extremely important for maintaining body iron homeostasis, where the protein HFE modulates *HAMP* induction [1, 2] and the induced peptide hepcidin regulates systemic iron homeostasis upon systemic iron elevation [17, 18]. However, the intracellular and extracellular iron-sensing mechanisms remain unclear and the upstream responses of the *HFE* mRNA to increasing extracellular and intracellular iron levels and its co-relation with *HAMP* mRNA have been rarely studied. Therefore, in this short study we aimed to discriminate between the effects of intracellular and extracellular iron-loading. Hence, we examined *HFE* and *HAMP* expressions under high extracellular iron levels, high intracellular iron levels, and simultaneously increased intracellular and extracellular iron levels by treating Wt and recombinant HepG2 cells with/without a range of increasing holo-Tf concentrations. Such studies will not only help in elucidating the iron-sensing mechanisms but also in understanding iron-acquisition in non-hereditary iron-excess conditions.

### *HFE* mRNA expression is responsive to excess extracellular and intracellular iron

In our knowledge, no study has yet reported the effect of increasing holo-Tf concentration or saturation on *HFE* mRNA levels. We report for the first time, that increasing extracellular holo-Tf concentration causes elevation in *HFE* mRNA expression in the Wt HepG2 cells (Fig. 1b). As this increase occurred in the absence of intracellular iron elevation (Fig. 1a), it could be attributed exclusively to the elevated extracellular holo-Tf concentrations, thereby demonstrating the responsiveness of *HFE* mRNA towards excess extracellular iron. Furthermore, high *HFE* mRNA expression in the absence of extracellular iron, but presence of high intracellular iron (as observed in untreated recombinant cells) can be attributed exclusively to the high intracellular iron content (Figs. 1a, 2a, 3a). This indicates the responsiveness of *HFE* mRNA exclusively to high intracellular content. Collectively, *HFE* mRNA expression showed independent sensitivity to extracellular and intracellular iron loading.

### *HAMP* mRNA expression and iron

In the Wt cells, elevation of *HAMP* expression following holo-Tf supplementation (Fig. 1c) is an expected response following an iron stimulus [17, 19, 20]. These elevations occurred in the absence of increased intracellular iron, indicating that an increase in extracellular iron was sufficient for the induction and a major increase in intracellular iron content was not necessary. Interestingly, its wavy pattern of expression over the increasing holo-Tf concentrations displayed a typical hormonal characteristic where increased levels of a stimulant (here, holo-Tf) may not lead to a directly proportional mRNA response. This is because, unlike cytokines, hormone-peptides are ‘premade’ and released from vesicles following a stimulus, like incase of insulin [21]. In the absence of extracellular iron (untreated cells), the high *HAMP* expression in recombinant cells (Fig. 3b), indicated that *HAMP* could be induced exclusively due to high intracellular iron content (Figs. 1a, 2a).

### Interrelationship between *HFE* and *HAMP* expression patterns

A correlation between the mRNA responses of *HFE* and *HAMP* over the increasing holo-Tf concentrations was envisaged. The Wt cells showed no co-relation between the patterns of their responses (Fig. 1b, c), probably reflecting the hormonal characteristic of hepcidin. Conversely, the recombinant cells showed similarities between the patterns of *HFE* and *HAMP* expressions (Fig. 2b, c). Data in the recombinant showed that under intracellular iron excess, only subtle extracellular iron elevation could elevate *HFE* and *HAMP* expressions and further increase in extracellular iron led to either repression or an unaltered effect. This implies that both these genes can be induced by an external iron stimulus to regulate iron homeostasis, but preferably in the absence of intracellular iron loading. This could be a reason for deregulated iron metabolism and insufficient hepcidin production in non-hereditary iron excess conditions that show both, systemic and cellular iron loading, despite the presence of functional alleles of these genes.

Further studies are required to elucidate these mechanisms to better understand the iron-sensing and iron-loading mechanisms; aiming to design therapeutic interventions for the non-hereditary iron-excess pathologies.

## Conclusion

In this short study, the independent effects of extracellular and intracellular iron on *HFE* and *HAMP* expressions were examined. *HFE* mRNA demonstrated independent responsiveness to elevated extracellular and intracellular iron content, suggesting its involvement in sensing both, extracellular and intracellular iron. Under combined intracellular and extracellular iron loading, *HFE* and *HAMP* expressions showed similar patterns and *HAMP* was induced only by low holo-Tf concentration, a scenario resembling iron excess pathologies.

## Electronic supplementary material

Below is the link to the electronic supplementary material.

Supplementary material 1 (DOC 73 KB)

## Abbreviations

EMEM: Eagle’s minimal essential medium
Holo-Tf: Holotransferrin
H: Hours

## Gene annotations

*HAMP*: Gene encoding hepcidin
*HFE*: Gene encoding haemochromatosis protein HFE
