# Supplementary material for: HFE mRNA expression is responsive to intracellular and extracellular iron loading: short communication
Source: Mol Biol Rep. 2017 Aug 24;44(5):399–403. doi: 10.1007/s11033-017-4123-2 (PMC5640751; doi:10.1007/s11033-017-4123-2)
Supplement: Supplementary file 1 — Supplementary material 1 (DOC 73 KB) [file 11033_2017_4123_MOESM1_ESM.doc]

***HFE* mRNA expression is responsive to intracellular and extracellular iron loading: short communication**

Supplementary Fig.1.

a) b)


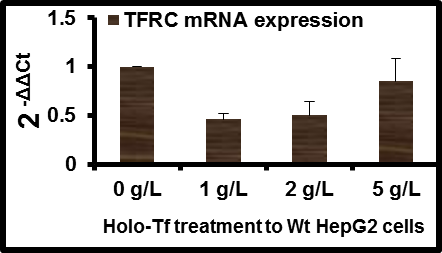

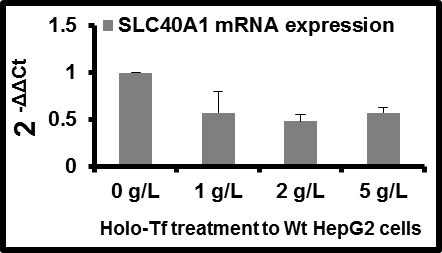


*

*

*

*

**Supplementary Fig.1 mRNA expression of iron-uptake and iron-efflux genes in Wt HepG2 cells**

Holo-Tf-induced mRNA expression of (a) *TFRC* (encoding transferrin receptor 1) and (b) *SLC40A1* (encoding ferroportin) in the Wt HepG2 cells have been shown. *p<=0.05 compared to untreated (0 g/L) controls. Data is presented as mean ± SEM (n=3).
